# Padé resummation of many-body perturbation theories

**DOI:** 10.1038/s41598-017-00355-w

**Published:** 2017-03-29

**Authors:** Y. Pavlyukh

**Affiliations:** 1 Department of Physics and Research Center OPTIMAS, University of Kaiserslautern, P.O. Box 3049, 67653 Kaiserslautern, Germany; 2 Institut für Physik, Martin-Luther-Universität Halle-Wittenberg, 06120 Halle, Germany

## Abstract

In a typical scenario the diagrammatic many-body perturbation theory generates asymptotic series. Despite non-convergence, the asymptotic expansions are useful when truncated to a finite number of terms. This is the reason for the popularity of leading-order methods such as the *GW* approximation in condensed matter, molecular and atomic physics. Appropriate truncation order required for the accurate description of strongly correlated materials is, however, not known *a priori*. Here an efficient method based on the Padé approximation is introduced for the regularization of perturbative series allowing to perform higher-order self-consistent calculations and to make quantitative predictions on the convergence of many-body perturbation theories. The theory is extended towards excited states where the Wick theorem is not directly applicable. Focusing on the plasmon-assisted photoemission from graphene, we treat diagrammatically electrons coupled to the excited state plasmons and predict new spectral features that can be observed in the time-resolved measurements.

## Introduction

Introduction of the Green’s function methods to electronic structure calculations is the most prominent achievement of the field-theoretic methods^1–3^ on par with the density functional theory having immediate technological applications^4, 5^. Even in the lowest (beyond the mean field) order one obtains significant improvements of e. g. the band gap through the correlation shifts (Δ). Including higher-order diagrams (vertex corrections) is numerically demanding and so far the truncation of perturbative expansions has been done in *ad hoc* manner. Having a tool to systematically perform higher-order self-consistent (*sc*) calculations would allow to make calculations for some representative systems and extrapolate these results in order to make quantitative statements on the convergence and accuracy of perturbative expansions for specific cases. However, there are fundamental obstacles on the way that arise from dealing with *diverging series* as the following consideration illustrates.

### Padé approximation

Let ${g}_{{\rm{model}}}(z)=1/(z-\epsilon -{\rm{\Delta }}-i\eta )$ be a model Green’s function (GF) and Δ be the energy shift due to some interaction. *g*_model_(*z*) can be expanded in terms of the non-interacting GF ${g}_{{\rm{model}}}^{\mathrm{(0)}}(z)=1/(z-\epsilon -i\eta )$ as a geometric series:

1 $${g}_{{\rm{model}}}(z)=\sum _{n=0}^{\infty }\frac{{{\rm{\Delta }}}^{n}}{{(z-\epsilon -i\eta )}^{n+1}}.$$

**Figure 1:**
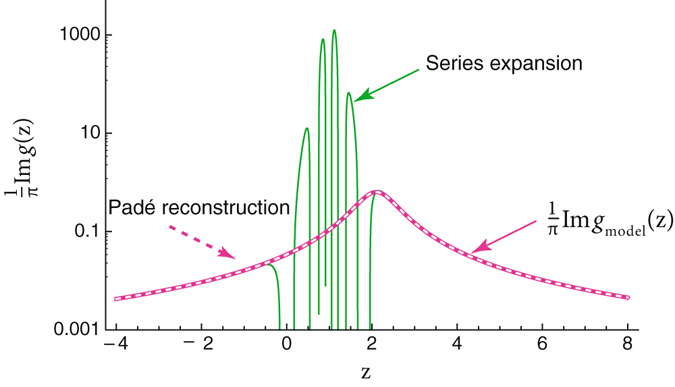
Reconstruction of *g*_model_(*z*) from its series expansion (1) in terms of ${g}_{{\rm{model}}}^{(0)}(z)$ using the Padé approximation (PA). Parameters are as follows: Δ = 1.1, $\epsilon$ = 1, *η* = 0.5. The series expansion (1) is restricted at *n*_max_ = 10, and PA is applied at the point *z* = 6. Notice that the original (magenta) and reconstructed (white) densities of states are practically indistinguishable.

The series expansion behaves oscillatory in the vicinity of the pole and approaches the original function at large *z*, i. e., for $|\frac{{\rm{\Delta }}}{z-\epsilon -i\eta }| < 1$. Nonetheless, a sensible spectral function, $A(z)=\frac{1}{\pi }{\rm{Im}}\,g(z)$, in the domain of interest can be reconstructed by using the Padé approximation. The procedure is outlined at Fig. 1 where the original function *g*_model_(*z*), the series expansion (1) and the Padé reconstruction are shown. The Padé approximation (PA) allows to obtain very accurate values also in the domain where the series (1) is divergent. The method works so well here because it is known in advance that GF consists of one pole only and this fact is used for the reconstruction: according to the exact form of *g*_model_(*z*) we use the [0/1] approximant (the Padé approximation has form of rational function denoted as [*M*/*N*]) with *M* + *N* + 1 coefficients, *M*, *N* are the orders of the numerator and denominator, respectively^6^). For realistic calculations we do not have this knowledge and have to rely on some additional assumptions about the analytic structure of the Green’s function. As an illustration let us consider the electron-boson Hamiltonian — a model which is ubiquitous in condensed matter physics.

## Methods

### Model specification

Consider a set of fermionic and bosonic quantum numbers and the associated creation and annihilation operators with standard commutation rules:

2 $${[{c}_{a},{c}_{b}^{\dagger }]}_{+}={\delta }_{ab},\quad {[{b}_{i},{b}_{j}^{\dagger }]}_{-}={\delta }_{ij}.$$

The model becomes non-trivial when a coupling between fermionic and bosonic degrees of freedom is introduced ${ {\mathcal H} }_{I}={\sum }_{ab}{\sum }_{i}{{\rm{\Gamma }}}_{ab}^{i}{c}_{a}^{\dagger }{c}_{b}{b}_{i}+{\rm{H}}{\rm{.c}}{\rm{.}}$ This very general model covers various physical scenarios: For instance, (i) the interaction of electrons in solids with *real* bosonic excitation such as phonons forming the basis of the polaron model (Sec 4.3 of Mahan^7^), novel applications include quantum dots coupled to nanomechanical oscillators^8^. (ii) Electronic excitations such as plasmons under some assumptions mediate the electron-electron interaction. This scenario was first introduced in the work of Lundqvist^9^ who considered coupling of the deep hole level to plasmonic excitations in metals with a well known analytic solution^10–12^. Another prominent example is the photoemission process where the photoelectron interacts with the density fluctuations of the target^13^. (iii) Auxiliary bosonic degrees of freedom is a mathematical trick used to treat a pure electronic Hamiltonian such as the mixed-valence Hamiltonian, i. e., large-*U* Anderson model (*slave*-*boson* approach)^14^.

Beside the Hamiltonian, the diagrammatic structure of a model is determined by the state of interest. For instance, the no-hole state is of relevance for the x-ray absorption in the Lundqvist model, while for the photoemission one considers a state with exactly one deep hole. At variance, the ground state of the large-*U* Anderson model is determined as a state in which the sum of boson and fermion occupation numbers at each site is unity. For this two-component fermionic model, very different diagrams (non-crossing approximation) are relevant^15^.

Consider now the electron-boson Hamiltonian in its simplest form also known as the *S*-model:

3 $$ {\mathcal H} =\epsilon {c}^{\dagger }c+c{c}^{\dagger }\gamma (b+{b}^{\dagger })+{\rm{\Omega }}{b}^{\dagger }b,$$

where *c* is the creation operator of the deep hole with energy $\epsilon$, *b* ^†^ is the bosonic creation operator of the plasmon with the energy Ω. The generalization to the case of multiple fermion kinds as in the mixed-valence impurity model or the plasmon dispersion is possible and will be commented on after the presentation of the diagrammatic solution. The Hamiltonian (3) is quite versatile and is applicable to other scenarios such as resonant-tunneling through a single level coupled to wide-band phonons^16^. Remarkably, also the two particle GF can be found analytically^17^; the model can be solved at finite temperatures, and some its non-equilibrium properties have been studied^18, 19^.

Let us consider the following Green’s function

$$g(t-t^{\prime} )=-i\langle {\psi }|T[c(t){c}^{\dagger }(t^{\prime} )]|\psi \rangle ,$$

where |*ψ*〉 is the exact ground state of the *no*-*hole system* with ${n}_{b}=\langle {b}^{\dagger }b\rangle =0$. It can be diagrammatically found by writing the cumulant expansion for the Green’s function $g(t)={g}^{(0)}(t){e}^{C(t)}$ in terms of non-interacting GF *g* ^(0)^(*t*) and the *cumulant function C*(*t*). Observing that only a single diagram contributes to the cumulant function (in a more general scenario it fulfills an integral equation^20, 21^) one writes

4 $$C(t)=-{(\frac{\gamma }{{\rm{\Omega }}})}^{2}(1+i{\rm{\Omega }}t-{e}^{i{\rm{\Omega }}t}).$$

**Figure 2:**
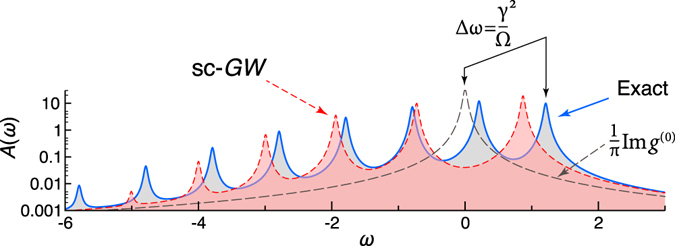
Spectral function at the following values of the parameters $\epsilon$ = 0, Ω = 1, *γ* = 1.1, *η* = 0.03 and different levels of theory: exact (full line), self-consistent first-order (short dashes), zeroth iteration (long dashes). Shaded areas are equal to unity.

**Figure 3:**
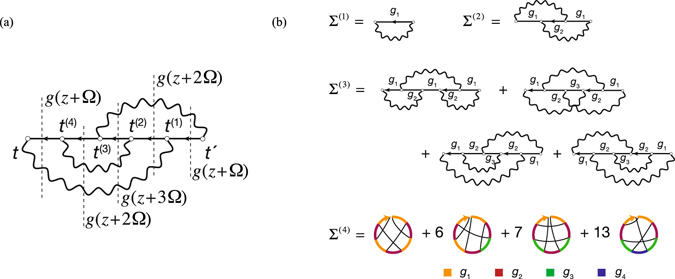
(**a**) Example of the self-energy in the time domain. The system contains only holes. Therefore, there is only one possible time-ordering $t < {t}^{(4)} < {t}^{(3)} < {t}^{(2)} < {t}^{(1)} < t^{\prime}$. Bosonic propagators are denoted as wavy-lines. In the frequency representation the given SE diagram yields ${\rm{\Sigma }}(\omega )={({\gamma }^{2})}^{3}g(\omega +{\rm{\Omega }})g(\omega +2{\rm{\Omega }})g(\omega +$ $3{\rm{\Omega }})g(\omega +2{\rm{\Omega }})g(\omega +{\rm{\Omega }})$. (**b**) Four lowest orders of the SE diagrammatic expansion of the electron-boson model. Notice that two diagrams of the third-order containing loops are not shown because they are equal to zero. The fourth-order self-energy is given in terms of chord diagrams with color-coding. Only one representative for each class is shown. Due to the absence of loops, an isomorphism between the Feynman diagrams and the chord diagrams can be established.

The corresponding exact spectral function is compared with the zeroth order and the self-consistent *GW* (sc-*GW*) approximations in Fig. 2. The latter is computed using the first diagram in Fig. 3(b); illuminating discussion of the *GW* approximation in electron-electron vs. electron-boson cases can be found in ref. 18. The results are plotted for the strongly correlated regime (*γ* > Ω) and can be characterized as follows: (i) The quasiparticle (QP) peak is shifted by the energy ${\rm{\Delta }}\omega =\frac{{\gamma }^{2}}{{\rm{\Omega }}}$ compared to the noninteracting case; (ii) this main peak is followed by the ladder of plasmonic satellites; (iii) the self-consistent *GW* method predicts the satellites. However, the position of even the main peak is wrong. This inaccuracy is the main motivation for performing higher-order diagrammatic calculations.

### Diagrammatic properties

Because the ground state is a no-hole state, ${c}^{\dagger }|{\psi }\rangle$ vanishes and, hence, the time-ordered Green’s function only consists of the hole propagator, $g(t-t{\rm{^{\prime} }})=\theta (t{\rm{^{\prime} }}-t){g}^{ < }(t-t{\rm{^{\prime} }})$, i. e., can be expressed solely in terms of the lesser GF component, and the corresponding non-interacting GF takes a form: ${g}^{(0)}(t-t^{\prime} )=i\theta (t^{\prime} -t){e}^{-i\epsilon (t-t^{\prime} )}$. This fact simplifies the diagrams considerably: (i) in the expansion of the Green’s function (*g*) and the self-energy (Σ) all intermediate points are time-ordered (Fig. 3(a)); (ii) diagrams containing loops necessarily yield a zero contribution (this is not the case for nonequilibrium states where renormalization of bosonic propagators by fermionic loops needs to be additionally considered^18, 22^). These properties allow to write the self-energy (SE) for this model in analytic form. Because there is no spatial degrees of freedom, the problem is similar to that of the Feynman diagrams enumeration which can be solved by collapsing the space-time variables to one point (the zero-dimensional model^23–26^).

Let ${{\rm{\Sigma }}}^{(n,\nu )}(\omega )$ be an *n* th-order self-energy term corresponding to a particular diagram, which will be denoted as *v*. We will prove below that the corresponding expression in the frequency representation is given by the product:

5 $${{\rm{\Sigma }}}^{(n,\nu )}(\omega )={({\gamma }^{2})}^{n}\prod _{i=1}^{2n-1}g(\omega +{k}_{i}^{(n,\nu )}{\rm{\Omega }}),$$

where ${k}_{i}^{(n,\nu )}$ is the integer number of absorbed plasmons in each fermionic line. Let us position 2*n* − 1 vertical lines such that they cut each fermionic line (Fig. 3(a)). Then ${k}_{i}^{(n,\nu )}$ is computed as a number of bosonic lines crossing *i* th vertical line. Equation (5) can be derived by using the nonequilibrium Green’s function (NEGF) formalism. Let a vertical line separate times lying on the forward and backward branches of the Keldysh contour in an expression for the *lesser* self-energy (Σ^<^). Consider, for instance, a third vertical line at Fig. 3(a). It contributes ${g}^{ < }(\omega -{y}_{1}-{y}_{2}-{y}_{3}){W}^{ < }({y}_{1}){W}^{ < }({y}_{2}){W}^{ < }({y}_{3})$ to Σ^<^(ω). Here, ${W}^{ < }(y)={\gamma }^{2}\delta (y+{\rm{\Omega }})$ is the lesser bosonic propagator. Performing three frequency integrals (over *y*_1_, *y*_2_, *y*_3_) a contribution proportional to *g* ^<^(ω + 3Ω) is obtained. Similar considerations can be repeated for each vertical line and fermionic propagator yielding in total 2*n* − 1 terms for each *n* th-order SE diagram ${{\rm{\Sigma }}}^{ < }(\omega )={\sum }_{i=1}^{2n-1}{f}_{i}(\omega ){g}^{ < }(\omega +{k}_{i}{\rm{\Omega }})$. Now, since *f*_*i*_(*ω*) are non-singular the generic expression for the time-ordered self-energy (5) is obtained.

Equation (5) serves as the starting point for numerics; complexity goes into the generation of Feynman diagrams and the determination of the coefficients ${k}_{i}^{(n,\nu )}$. Together with the Padé approximation this is the second important ingredient of our approach. The coefficients are computed purely algebraically by introducing an external time-dependent potential *ϕ*(1) (for brevity time variables are denoted as ${t}_{i}\equiv i$) and using the variational derivative technique^27^ as in the derivation of Hedin’s equations^28^. As was shown above the bosonic propagator in the present model does not renormalize (loops give zero contribution), i. e., $\frac{\delta W(12)}{\delta \varphi \mathrm{(3)}}=0$, leading to a simpler set of equations:

6 $${\rm{\Gamma }}(12,3)=\delta (12)\delta (13)+\frac{\delta {\rm{\Sigma }}(12)}{\delta V(3)},$$

7 $${\rm{\Sigma }}(12)=i\int W(13)g(14){\rm{\Gamma }}(42,3)d(34),$$

8 $$\frac{\delta g(12)}{\delta V(3)}=\int g(14)g(52){\rm{\Gamma }}(45,3)d(45),$$

where Γ(12, 3) is the vertex function, Σ(12) is the electron self-energy, and *V*(3) is the external plus the induced field in the system. All these quantities are functionally dependent on the external field *ϕ*(3) and on the full electron propagator *g*(12). The set of equations (6, 7 and 8) can now be iterated starting from ${{\rm{\Gamma }}}^{0}(12,3)=\delta (12)\delta (13)$ leading to the diagrams shown at Fig. 3(b).

**Figure 4:**
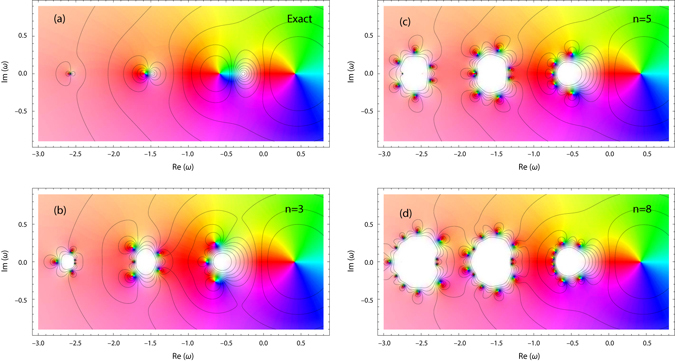
Electron Green’s function *g*(*ω*) resolved in complex plane for Ω = 1 and *γ* = 0.65. Approximations are computed using SE of orders 3 (**b**), 5 (**c**), and 8 (**d**) and compared to the exact solution (**a**). For *g*(*ω*), the hue channel represents the arg[*g*(*ω*)], the saturation channel represents the modulus |*g*(*ω*)|, and the contour plot depicts tanh |*g*(*ω*)|.

The chord diagram^29, 30^ representation is natural in this case because according to the analysis above the fermionic loops yield zero contribution. In order to further facilitate the interpretation of the graphs in frequency space we use color coding for the coefficients ${k}_{i}^{(n,\nu )}$ entering the GF arguments. The graphs were generated by our symbolic algorithm in mathematica computer algebra system. Conversion from the time to the frequency domain is likewise performed using a symbolic algorithm. The self-energy which is accurate to the sixth order comprises 1, 1, 4, 27, 248, and 2830 diagrams of the first to sixth orders, respectively, has the following algebraic representation (see Supplementary Information for higher order terms):

9 $$\begin{array}{rcl}{\rm{\Sigma }}[g] & = & {\gamma }^{2}{g}_{1}+{({\gamma }^{2})}^{2}{g}_{2}{g}_{1}^{2}+{({\gamma }^{2})}^{3}({g}_{2}^{2}{g}_{1}^{3}+3{g}_{2}^{2}{g}_{3}{g}_{1}^{2})+{({\gamma }^{2})}^{4}({g}_{2}^{3}{g}_{1}^{4}+6{g}_{2}^{3}{g}_{3}{g}_{1}^{3}\\  &  & +7{g}_{2}^{3}{g}_{3}^{2}{g}_{1}^{2}+13{g}_{2}^{2}{g}_{3}^{2}{g}_{4}{g}_{1}^{2})+{\mathscr{O}}({({\gamma }^{2})}^{5}),\end{array}$$

where ${g}_{k}\equiv g(\omega +k{\rm{\Omega }})$. Our explicit form for the self-energy dictates that the singularities of Σ(*ω*) should be located exactly at the GF poles. It is physically wrong as it is well known that the SE poles lie between the poles of the corresponding exact Green’s function^31^. These two facts can be reconciled by noticing that already starting with the second order term ${({\gamma }^{2})}^{2}g(\omega +{\rm{\Omega }})g(\omega +2{\rm{\Omega }})g(\omega +{\rm{\Omega }})$ the self-energy contains *higher*-*order* poles. As in our toy model (Fig. 1) they are responsible for the energy shift. However, the convergence is mathematically more intricate. In Fig. 4 the exact Green’s function is compared with its reconstruction using SE of different orders (non-self-consistent calculation with Σ[*g*] being a functional of exact *g*). With increasing the perturbative order, the higher-order poles accumulate on the edges of lagoons (which enclose true simple poles) where the Green’s function is not properly represented (vanishing real and imaginary parts). Outside the lagoons, the perturbative series converge rather rapidly as can be seen from the contour plots of tanh |*g*(*ω*)|. As in the toy model, PA can be used to obtain the Green’s function in the whole complex plane. This will be illustrated now by *sc* calculations for the electron-boson model at equilibrium and at zero temperature.

## Results

### Self-consistent calculations at equilibrium and at *T* = 0

**Figure 5:**
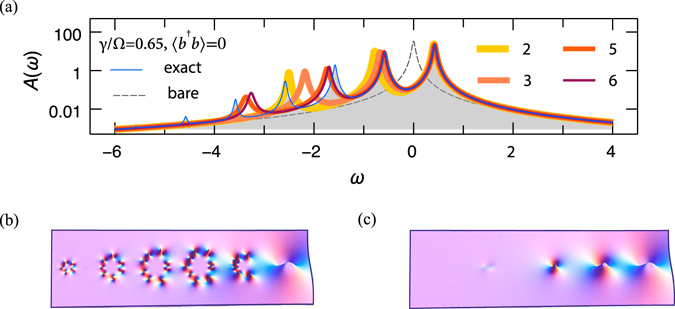
(**a**) Spectral function of the electron-boson model at different levels of theory (perturbative orders *n* = 2, …, 6 are denoted by different line styles) for the following values of parameters: $\epsilon$ = 0, Ω = 1, *γ* = 0.65, *η* = 0.03. The effect of Pade regularization is depicted in the two panels below: (**b**) no PA is applied, the higher-order poles are visible; (**c**) the regularized spectral function contains only simple poles.

Assume that in the course of a self-consistent calculation an approximate GF (*g* ^(*i*)^(*ω*)) has been obtained. Using the diagrammatic expansion (9) we evaluate the self-energy ${\rm{\Sigma }}[{g}^{(i)}]({\omega }^{\ast })$ at a chosen frequency point. The point *ω* ^*^ should belong to the domain of convergence. In order to obtain the self-energy in the vicinity of the Green’s function poles where the series diverges (note the unphysical multiple poles in the complex *ω*-plane on Fig. 5(b)), we perform the Padé approximation ${\rm{\Sigma }}[{g}^{(i)}]({\omega }^{\ast })\to {\tilde{{\rm{\Sigma }}}}^{(i)}(\omega )$ and use the new self-energy in the Dyson equation ${g}^{(i+1)}(\omega )={[\omega -\epsilon -{\tilde{{\rm{\Sigma }}}}^{(i)}(\omega )]}^{-1}\mathrm{.}$ Iterations are started from the noninteracting GF ${g}^{\mathrm{(0)}}(\omega )={(\omega -\epsilon -i\eta )}^{-1}$ and typically converge within some tens of cycles. Convergence is improved by using PA of variable order: on the first iteration cycle the non-interacting GF is used as an input leading to relatively simple self-energy that can regularized using PA of low order ([0/1]). In the course of *sc* calculations GF develops more satellites which require higher order PA (typically [11/12]) in order to accurately represent the self-energy. The quality of the resulting spectral function (cf. Fig. 5(b,c)) strongly depends on the order of perturbative expansions and on the electron-plasmon interaction strength *γ*. For the weakly correlated regime ($\gamma \simeq 0.2{\rm{\Omega }}$, we will show below that such a coupling strength is typical for monolayer graphene) already the *GW* approximation faithfully reproduces the exact spectral function, but it ceases to be valid in the *correlated regime* as demonstrated in Fig. 2. The energy of the QP peak is the major discrepancy. For γ = 0.65 (this value would be typical for the valence electrons in Al, Cu, Au metals), the third-order treatment substantially improves its position and strength, Fig. 5(a). Yet, the first satellite, which has a rather large contribution to the density of states at this value of *γ* (notice the logarithmic scale), represents a considerably more complicated feature. It can only be captured with a self-energy that is accurate to the 6th order (thin dark red line). However, even 3111 diagrams are not sufficient to reproduce the second-order satellite! There are known examples of GF calculations performed with even much larger number of diagrams, such as in diagrammatic quantum Monte Carlo^32^ study of the Fröhlich polaron^33^, Anderson^34^ and Hubbard^35^ models. The major distinction of our approach is that it operates with *skeletonic* (in terms of dressed propagators from *sc* calculations) expansions. SE expansions in terms of *bare* propagators are reviewed by Cini and D’Andrea^12^. They can be represented in terms of continued fractions.

### Estimates for realistic systems

Being able to perform higher order self-consistent diagrammatic calculations with high accuracy allows us to make predictions about the convergence of many-body perturbation theory (MBPT) for real-world systems, which is the main goal of this work. To this end, all relevant parameters for the mapping onto the electron-boson model need to be determined. Since $\epsilon$ barely shifts the electronic spectrum, there is only one relevant dimensionless parameter *a* = *γ*/Ω; in its terms the correlation shift is given by Δ$\epsilon$ = *a* ^2^Ω. The *a*-parameter for the hole states can be obtained by comparing the spectral strength of SE in the vicinity of $\epsilon$ − Ω and $\epsilon$ − 2Ω poles and be expressed in terms of the corresponding residues of the first and second order lesser self-energies (for the particle sector, similar expressions can be written in terms of the greater self-energies):

10 $${a}^{2}=\frac{{M}^{(2)}}{{M}^{(1)}}=\frac{{{\rm{r}}{\rm{e}}{\rm{s}}}_{\omega =\epsilon -2{\rm{\Omega }}}{{\rm{\Sigma }}}^{(2)}(\omega )}{{{\rm{r}}{\rm{e}}{\rm{s}}}_{\omega =\epsilon -{\rm{\Omega }}}{{\rm{\Sigma }}}^{(1)}(\omega )}=\frac{{\int }_{-{\rm{\infty }}}^{{\rm{\infty }}}-i\,{{\rm{\Sigma }}}^{(2), < }(\omega ){\rm{d}}\omega }{{\int }_{-{\rm{\infty }}}^{{\rm{\infty }}}-i\,{{\rm{\Sigma }}}^{(1), < }(\omega ){\rm{d}}\omega }.$$

The second form in terms of the zeroth spectral moments of the lesser self-energies is preferable for realistic systems, where due to the momentum dispersions of electrons and plasmons the SE singularities are partially smeared out. As a paradigmatic system we consider here the homogeneous electron gas (HEG)^3^. It is believed to capture main electronic properties of simple metals, is a prototypic system for deriving approximations for the exchange-correlation functional^36^ and has been widely studied using a variety of approaches^37–42^. Comparing with the electron-boson model, HEG is a considerably more complicated system: in addition to the excitations of multiple plasmon branches^43, 44^, the electron-electron interaction is also accompanied by the excitation of particle-hole (*p* − *h*) pairs. These effects are more pronounced for the states close to the Fermi sphere (*k* = *k*_*F*_). At the band bottom (the momentum *k* = 0) the phase-space for the excitation of *p* − *h* pairs is reduced and the mapping onto the electron-boson model is more justified. Therefore, this case will be considered here selecting (in conformance with the electron-boson model) only the SE diagrams describing the scattering processes with generation of plasmons. Such a selection is possible using the methods developed in refs 45 and 46. In particular it means that in the second order (in the screened Coulomb interaction) only  has to be considered (pluses and minuses here denote the position of time-arguments on the Keldysh contour: −/+ for forward/backward branches). After analytically performing the frequency integrations over *ω* and over the internal frequencies we are left with the following momentum integrals:

11 $${M}^{(1)}=2\alpha {r}_{s}{\int }_{0}^{{q}_{c}}{\rm{d}}y\,{n}_{{\rm{F}}}(y)\frac{t(y){\omega }_{pl}^{2}}{{\omega }_{pl}(y)},$$

12 $${M}^{\mathrm{(2)}}=\frac{2{\alpha }^{2}{r}_{s}^{2}}{{(2\pi )}^{3}}\int \frac{{{\rm{d}}}^{3}{{\bf{y}}}_{1}}{{y}_{1}^{2}}\int \frac{{{\rm{d}}}^{3}{{\bf{y}}}_{2}}{{y}_{2}^{2}}{n}_{{\rm{F}}}({y}_{3})\frac{t({y}_{1}){\omega }_{pl}^{2}}{{\omega }_{pl}({y}_{1})({y}_{2}^{2}-{y}_{3}^{2}+{\omega }_{pl}({y}_{1}))}\frac{t({y}_{2}){\omega }_{pl}^{2}}{{\omega }_{pl}({y}_{2})({y}_{2}^{2}-{y}_{3}^{2}+{\omega }_{pl}({y}_{2}))},$$

with ${y}_{3}=|{{\bf{y}}}_{1}+{{\bf{y}}}_{2}|,\,{\omega }_{pl}\equiv {\omega }_{pl}(0)=4\sqrt{\alpha {r}_{s}/(3\pi )}$ the classical plasmon frequency, and ${n}_{F}(y)=\theta (1-y)$ the Fermi distribution function. The momentum integrations are performed up to the value of critical momentum *q*_*c*_, i. e. the momentum at which the plasmonic oscillator strength $0\le t(y)\le 1$ vanishes. Equations (11 and 12) are exact, they show a typical scaling of perturbative expansions ${(\alpha {r}_{s})}^{n}$, where $\alpha ={(4/9\pi )}^{\mathrm{1/3}}$ and *r*_*s*_ is the Wigner-Seitz radius (in atomic units). The six-dimensional integration in *M* ^(2)^ can be somewhat simplified in spherical coordinates and reduced so to a 4d integral amenable to the Monte-Carlo approach introduced in ref. 47 and applied in refs 45, 46 and 48 to more complicated problems. Nonetheless it is instructive to introduce an approximation with the goal of estimating the *a*-parameter analytically.

We focus on the limit of large densities, $\alpha {r}_{s}\to 0$, where random phase approximation holds. In this case the critical momentum likewise approaches zero and we set $t(y){\omega }_{pl}^{2}/{\omega }_{pl}(y)\approx {\omega }_{pl}$ in the first integral leading to ${M}^{(1)}\approx 2\alpha {r}_{s}{\omega }_{pl}{q}_{c}$. In the second integral ${y}_{1,2}^{2}-{y}_{3}^{2}\ll {\omega }_{pl}({y}_{2,1})$ and we likewise neglect the plasmon dispersion resulting in ${M}^{(2)}\approx 2{\alpha }^{2}{r}_{s}^{2}{(4\pi {q}_{c})}^{2}/{(2\pi )}^{3}$ or

13 $$a=\sqrt{\frac{2\alpha }{\pi }\frac{{r}_{s}{q}_{c}}{{\omega }_{pl}}}.$$

**Figure 6:**
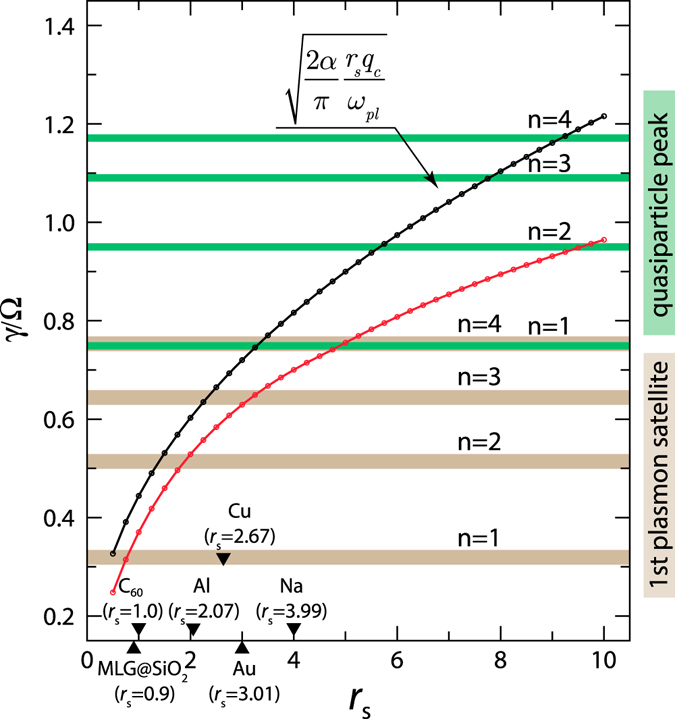
Electron-plasmon coupling as a function of the Wigner-Seitz radius *r*_*s*_. Red curve represents the numerical results obtained by the integration of Eqs (11 and 12), black curve denotes the analytical approximation. On the *x*-axis materials and their electron densities are additionally depicted. Horizontal lines denote values of *a* at which positions of main *qp*-peak and first plasmon satellite are obtained with at least 10% accuracy using the *n* th-order *sc* theory.

Our numerical calculations (Fig. 6) confirm that despite a number of approximations the analytical estimate holds well in the weakly correlated limit. In the opposite limit the phase-space restriction due to *n*_*F*_(*y*_3_) and the plasmonic dispersion gain significance reducing the electron-plasmon coupling as compared to the estimate (13).

Having the electron-plasmon interaction strength at our disposal, we can make some concrete predictions about the perturbative order required to accurately describe certain features in the electronic spectrum. As such, let us consider here the position of *qp*-peak and its first plasmon satellite. The horizontal lines in Fig. 6 denote the values of *γ*/Ω at which the *sc* theory yields these features with 10% accuracy. For instance, already the second-order self-energy is sufficient to describe both features in Al, whereas for Na 4th-order diagrams would be required to correctly compute the energetic position of the first plasmonic satellite! In contrast to these simple metals, the 2d systems are much more diverse in terms of their electron density parameter *r*_*s*_. By changing the substrate or by doping the system, it is possible to tune *r*_*s*_ from the weakly correlated limit, such as in the case of monolayer graphene (for MLG *r*_*s*_ ≈ 2.2/${\epsilon }_{M}$, ${\epsilon }_{M}$ is the background dielectric constant), to a strongly correlated regime in, e. g., 2DEG in GaAs where for the carrier concentration of *n* ≈ 10^9^ cm^2^ the density *r*_*s*_ ≈ 13 has been reported^45^ (${r}_{s}=4/\sqrt{\bar{n}}$, $\bar{n}=n/{10}^{10}{{\rm{cm}}}^{-2}$). At even higher densities a transition to the Wigner crystal phase takes place^46^. Our analysis (Fig. 6) thus justifies the use of *GW* approximation for the monolayer graphene^47, 48^. Similar conclusion holds for fullerenes (*r*_*s*_ ≈ 1.0), which are molecules of graphene wrapped by the introduction of pentagons on the hexagonal lattice, endorsing the use of *GW* for these systems^49, 50^.

As can be seen from Fig. 6, with increasing *r*_*s*_ the skeletonic diagrammatic SE expansion quickly becomes impractical. Therefore, different resummation methods such as *sc* parquet approximation (see Supplementary Information) need to be used.

## Self-consistent calculations for excited states

**Figure 7:**
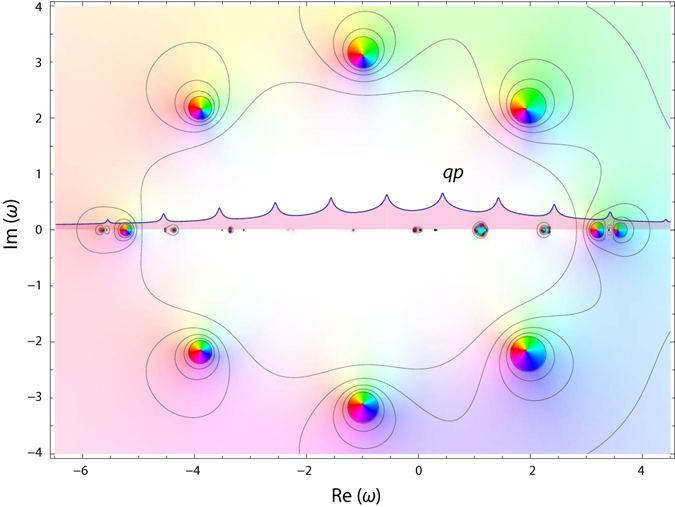
Electron Green’s function *g*_*β*_(*ω*) resolved in complex plane for Ω = 1, *γ* = 0.65 and *n*_*b*_ = 1/2 computed using the forth order SE. Color coding as in Fig. 4. Exact spectral function is superimposed.

So far results at zero temperature and zero bosonic occupation number have been presented. Equilibrium finite temperature scenario seems to be an obvious extension because the Wick theorem still holds^51^. Quite unexpectedly, calculations indicate that *sc* approach can only be realized at the lowest order, i. e., at the level of *GW* approximation. Higher-order self-energies cannot be regularized with the help of PA. To understand this behavior, it is instructive to analyze the exact electron Green’s function for a *finite boson occupation number* ${n}_{b}=\langle {b}^{\dagger }b\rangle ={(\exp (\beta {\rm{\Omega }})-1)}^{-1} > 0$. A solution in terms of a continued fraction is known due to Cini^52^, but it also represent no difficulty to generalize the cumulant function (4) to this case. Using the standard finite temperature expression ${W}^{ < }(y)={\gamma }^{2}(({n}_{b}+1)\delta (y+{\rm{\Omega }})+{n}_{b}\delta (y-{\rm{\Omega }}))$ we obtain

14 $${g}_{\beta }(\omega )=\sum _{n=0}^{{\rm{\infty }}}\frac{{a}^{2n}}{n!}\exp (-{a}^{2}(2{n}_{b}+1))\sum _{k=0}^{n}\frac{(\begin{array}{c}n\\ k\end{array}){({n}_{b}+1)}^{k}{n}_{b}^{n-k}}{\omega -\epsilon -{\rm{\Omega }}{a}^{2}+{\rm{\Omega }}(2k-n)-i\eta },$$

where as above $a=\frac{\gamma }{{\rm{\Omega }}}$, and $$(\begin{array}{c}n\\ k\end{array})$$ is the binomial coefficient, and the averaged boson occupation number is *n*_*b*_. The poles are now situated on both sides of the *qp*-peak. In addition to the poles associated with the *excitation* of bosons, there are poles associated with the energy *absorption* from the thermally excited bosons. Thus, real *ω* ^*^ points cannot be used for the Padé approximation because they are inevitably situated in the proximity of poles. In fact, by plotting an approximate GF in the complex plane (Fig. 7) we see that the situation cannot be cured even by shifting *ω* ^*^ away from the real axis: the lagoon that encompasses regions of non-convergence is extended along real and imaginary axes of the complex plane posing problems for the regularization.

In thermal equilibrium at finite *β* (the inverse temperature) the mixed bosonic state is represented by the density matrix $\rho (\beta )=(1-\exp (-\beta {\rm{\Omega }}))\exp (-{n}_{b}\beta {\rm{\Omega }})$. It is of interest to find the electron Green’s function for *pure* bosonic states corresponding to a given *n*_*b*_. These states are particularly relevant for the state-of-the-art ultrafast experiments^53, 54^ where the interaction with laser pulses cannot be assumed to follow the adiabatic path^22, 55, 56^. Excitation of confined plasmons in graphene by impinging free electrons^57^ can be considered as a paradigmatic system. The analysis of García de Abajo suggests that 100 eV electrons interacting with a monolayer graphene excite on average one plasmon per electron. This is a sizeable effect that can be detected with standard spectroscopic methods as discussed below. To describe photoemission from the system excited by impinging electrons, we compute the single-particle GF for *n*_*b*_ > 0 using the Feynman disentangling of operators (see Supplemental Information). Expansions of *g* ^[*nb*]^(*ω*) in terms of the shifted *n*_*b*_ = 0 propagators ${g}^{[0]}\equiv g(\omega )$ provide an exact solution for the particular case of electron-boson model (3). However, perturbative *sc* calculations using some approximations for the electron SE have additional advantage that they can be generalized to more complicated scenarios, e. g. include dispersion and multiple electronic and bosonic bands. Let us recall that perturbative expansions of correlators in MBPT (including the self-energies) are generated by expanding the contour evolution operator $\hat{{\mathscr{T}}}{e}^{-i{\int }_{-{\rm{\infty }}}^{{\rm{\infty }}}d\tau {{\mathscr{H}}}_{I}(\tau )}$ in powers of the interaction and expressing averages of the operator products in terms of products of simple propagators (the Wick theorem). For the ground state (generalizations to arbitrary initial states are also possible^51^) of a system of fermions the time-ordered product of any number of field operators splits up into the sum of the products of normal products of pairs, the averages of normal products being equal to zero. For bosonic systems with *n*_*b*_ > 0 a straightforward generalization of Eq. (9) would not work since it relies upon the Wick theorem, which cannot be formulated here because averages of the normal product of bosonic operators are non-zero^58^. Nonetheless a method to generate *excited states self*-*energies* can be devised.

The method can be illustrated by considering the computation of bosonic averages, ${\langle Q({t}_{1})Q({t}_{2})\ldots Q({t}_{n})\rangle }^{[{n}_{b}]}$, over the states with fixed particle number, where square brackets are used to distinguish the state with fixed boson number from the thermal state. As a particular example, *n*_*b*_ = 1 will be computed in accordance with the scenario of plasmons in graphene excited by means of impinging electrons. Recalling that bosonic displacement operator is given by $Q(t)=\frac{1}{\sqrt{2}}(b{e}^{-i{\rm{\Omega }}t}+{b}^{\dagger }{e}^{i{\rm{\Omega }}t})$ one can write:

15 $${\langle \hat{{\mathscr{T}}}Q({t}_{1})Q({t}_{2})\ldots Q({t}_{n})\rangle }^{[1]}=\mathop{\mathrm{lim}}\limits_{\tau \to 0}\langle \hat{{\mathscr{T}}}Q(-\tau )Q({t}_{1})Q({t}_{2})\ldots Q({t}_{n})Q(\tau )\rangle ,$$

where $\hat{{\mathscr{T}}}$ is the contour ordering operator, and for the computation of the correlator on the right hand side we first set $-\tau \,\prec \,{t}_{1},\ldots {t}_{n}\,\prec \,\tau$ (where $\prec$ is the order relation with respect to $\hat{{\mathscr{T}}}$) and subsequently evaluate the limit $\tau \to 0$. Now, on the right hand side we have a *n*_*b*_ = 0 correlator which can be computed using the standard Wick theorem. The procedure is also suitable for the computation of the electron self-energy because the conditions of validity of the Wick theorem for fermionic degrees of freedom are not affected by the choice of a reference bosonic state. By computing corresponding bosonic correlators we first arrive at the SE being a functional of bare GFs (${{\rm{\Sigma }}}^{[1]}={\bar{{\rm{\Sigma }}}}_{r}[{g}^{(0)}]$, see Supplemental Information for the explicit form), and by iterating the Dyson equation further express the self-energy in terms of the full propagators ${{\rm{\Sigma }}}^{[1]}=\bar{{\rm{\Sigma }}}[{g}^{[1]}]$. This leads to a generalization of Eq. (9) for the *n*_*b*_ = 1 pure bosonic state:

16 $$\begin{array}{rcl}\bar{{\rm{\Sigma }}}[g] & = & {\gamma }^{2}({g}_{-1}+2{g}_{1})+{({\gamma }^{2})}^{2}(2{g}_{1}^{2}{g}_{2}-2{g}_{0}{g}_{1}^{2}-2{g}_{0}{g}_{-1}^{2}-{g}_{-2}{g}_{-1}^{2})\\  &  & +{({\gamma }^{2})}^{3}(3{g}_{-2}^{2}{g}_{-1}^{3}+6{g}_{0}^{2}{g}_{-1}^{3}+4{g}_{-2}{g}_{0}{g}_{-1}^{3}+{g}_{-3}{g}_{-2}^{2}{g}_{-1}^{2}-2{g}_{0}^{2}{g}_{1}{g}_{-1}^{2}\\  &  & +2{g}_{0}^{2}{g}_{1}^{2}{g}_{-1}+6{g}_{0}^{2}{g}_{1}^{3}-4{g}_{0}{g}_{1}^{3}{g}_{2}+8{g}_{1}^{2}{g}_{2}^{2}{g}_{3})+{\mathscr{O}}({({\gamma }^{2})}^{4}).\end{array}$$

In contrast to the *n*_*b*_ = 0 case, SE in terms of the dressed propagators given by the equation above possesses less economical series than ${\bar{{\rm{\Sigma }}}}_{r}[{g}^{(0)}]$. This can be understood by considering the lowest order Σ^[1]^ which is identical for the nonequilibrium and thermal states (latter can be inferred from the first term of Equation (9) using ${g}_{1}\to 2{g}_{1}+{g}_{-1}$ for *n*_*b*_ = 1) and which has identical expressions in terms of bare and full propagators. For *n*_*b*_ = 1, the single-shot calculation yields a single peak above the *qp* state, however, multiple satellites above the *qp* state are created if the procedure is iterated to self-consistency. All of them but one quickly diminish when higher-order SE terms are included. The cancellation is achieved owing to extra terms containing *g*_−1_ (*i* > 1); they do not appear in ${\bar{{\rm{\Sigma }}}}_{r}[{g}^{(0)}]$.

**Figure 8:**
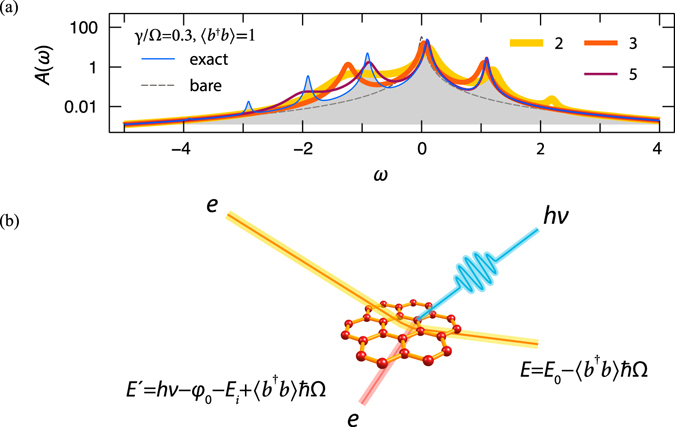
(**a**) Spectral function of the system with *n*_*b*_ = 1 at different levels of theory (perturbative orders *n* = 2, 3, 5 are denoted by different line styles) for the following parameters: $\epsilon$ = 0, Ω = 1, *γ* = 0.3, *η* = 0.03. Single plasmon satellite to the right of *qp* is a unique feature of this excited bosonic state. It can experimentally be observed in a ultrafast experiment sketched in panel (**b**).

Corresponding *sc* results are obtained using the Padé approximation, Fig. 8(a). The extra satellite at $\varepsilon +\frac{{\gamma }^{2}}{{\rm{\Omega }}}+{\rm{\Omega }}$, is a marked feature of the spectrum that can be observed in time-resolved photoemission. A possible scenario for such an experiment is depicted in Fig. 8(b): as in the proposal of García de Abajo^57^ impinging free electrons excite the bosonic subsystem — plasmons. The same effect can, in principle, be achieved with ordinary laser pulses, however, for confined systems, direct optical excitation of plasmons is less efficient. On the second step, optical or UV pulse detects the change in the electronic density of states provided the delay between the electron pump and the photon probe does not exceed the plasmon relaxation time. Thus, the proposed experiment is capable of directly measuring the electron-plasmon interaction strength and, if resolved in time, yielding the plasmon relaxation time.

## Discussion

There is more than a computational complexity which prevents the applications of the MBPT beyond the leading order. The resulting asymptotic series lead to Green’s functions with incorrect physical properties such as non-positive densities and higher-order poles already at the second order^59–61^. Besides the interaction strength, the domain of convergence strongly depends on the microscopic details of the model: continuous space vs. lattice formulation^62^ and also on the temperature as discussed below. For various statistical and many-body models PA has been used to extend perturbative expansions beyond their domain of convergence^63–66^. The same mathematical approach is used here in a different context, to regularize the electron SE. Using NEGF formalism the self-energy of the electron-boson model is derived in an explicit form for the ground *n*_*b*_ = 0 (Equation 9) and excited *n*_*b*_ = 1 (Equation 16) bosonic states and a connection of its diagrammatic expansion to a certain class of chord diagrams is demonstrated. With the help of the developed symbolic algorithm higher-order self-consistent calculations were performed, Figs 5 and 8. More complicated models can be treated starting from the same idea and applying the momentum average approximation^67^, which was proved to be accurate for the description of dressed particles in the Holstein polaron model^68^.

To achieve the main goal of this work and make quantitative predictions on the convergence of MBPT for realistic systems, we map the homogeneous electron gas onto the studied model. It allows to express our finding in terms of the density parameter *r*_*s*_ (Fig. 6). We find that the number of self-energy terms needed to be included in the *sc* calculations grows rapidly with *r*_*s*_: while for *r*_*s*_ = 1 (typical for Carbon materials) the first order accurately describes the first plasmon satellite, even the third order SE is not sufficient at metallic densities (*r*_*s*_ = 4).

Finally, extension of the formalism towards electron-boson systems in an excited bosonic state represents an interesting conceptual problem and is relevant for the description of ultrafast spectroscopic experiments. For thermal states at finite temperature, there is a clear mathematical argument precluding self-consistent calculations. However, we demonstrate, the calculations can be performed for pure states with finite boson occupation number (*n*_*b*_ > 1). A method is derived, allowing to obtain perturbative expansions even though the Wick theorem is not applicable in this case. For *n*_*b*_ = 1, our calculations show additional plasmonic satellite above the quasiparticle peak. Even at rather low electronic density, such as found in graphene, the modification of the spectral function compared to the *n*_*b*_ = 0 state is substantial and can be experimentally observed. However, for confined systems^69^, direct optical excitation of plasmons is inefficient^70, 71^. Therefore, we argue, by exciting the system using a beam of free electrons, the appearance of extra satellites in the electronic density of states can be probed by the time-resolved photoemission^22, 72, 73^. This setup allows to directly measure the electron-plasmon interaction strength and the plasmon relaxation time.

## Electronic supplementary material

Supplementary information
