# Erratum: Padé resummation of many-body perturbation theories

**DOI:** 10.1038/s41598-017-07624-8

**Published:** 2017-08-21

**Authors:** Y. Pavlyukh

**Affiliations:** 1 Department of Physics and Research Center OPTIMAS, University of Kaiserslautern, P.O. Box 3049, 67653 Kaiserslautern, Germany; 2 Institut für Physik, Martin-Luther-Universität Halle-Wittenberg, 06120 Halle, Germany

*Scientific Reports* **7**:504; doi:10.1038/s41598-017-00355-w; Article published online 29 March 2017

An incorrect version of the Supplementary Information file was inadvertently published with this Article. The correct Supplementary Information file now accompanies this Article.

