# Supplementary material for: Padé resummation of many-body perturbation theories
Source: Sci Rep. 2017 Mar 29;7:504. doi: 10.1038/s41598-017-00355-w (PMC5428253; doi:10.1038/s41598-017-00355-w)
Supplement: Supplementary file 1 — Supplementary information [file 41598_2017_355_MOESM1_ESM.pdf]

Padé resummation of many-body perturbation theories

Y. Pavlyukh[a)]

(Dated: 23 December 2016)

I. ELECTRON-BOSON MODEL IN THE GROUND BOSONIC STATE

Using the algebraic method from Sec. I of the main text the electron self-energy can be written in terms of shifted dressed propagators as follows:

$$\begin{aligned}\Sigma[g] = {}& \gamma^2 g_1 + (\gamma^2)^2 g_2 g_1^2 + (\gamma^2)^3 \left(g_2^2 g_1^3 + 3 g_2^2 g_3 g_1^2\right) + (\gamma^2)^4 \left(g_2^3 g_1^4 + 6 g_2^3 g_3 g_1^3 + 7 g_2^3 g_3^2 g_1^2 + 13 g_2^2 g_3^2 g_4 g_1^2\right)\\
&+ (\gamma^2)^5 \left(g_2^4 g_1^5 + 9 g_2^4 g_3 g_1^4 + 23 g_2^4 g_3^2 g_1^3 + 26 g_2^3 g_3^2 g_4 g_1^3 + 15 g_2^4 g_3^3 g_1^2 + 58 g_2^3 g_3^3 g_4 g_1^2 + 45 g_2^2 g_3^3 g_4^2 g_1^2 + 71 g_2^2 g_3^2 g_4^2 g_5 g_1^2\right)\\
&+ (\gamma^2)^6 \big(g_1^6 g_2^5 + 12 g_1^5 g_2^5 g_3 + 48 g_1^4 g_2^5 g_3^2 + 72 g_1^3 g_2^5 g_3^3 + 31 g_1^2 g_2^5 g_3^4 + 39 g_1^4 g_2^4 g_3^2 g_4 + 194 g_1^3 g_2^4 g_3^3 g_4 + 183 g_1^2 g_2^4 g_3^4 g_4\\
&+ 90 g_1^3 g_2^3 g_3^3 g_4^2 + 313 g_1^2 g_2^3 g_3^4 g_4^2 + 145 g_1^2 g_2^2 g_3^4 g_4^3 + 142 g_1^3 g_2^3 g_3^2 g_4^2 g_5 + 310 g_1^2 g_2^3 g_3^3 g_4^2 g_5 + 470 g_1^2 g_2^2 g_3^3 g_4^3 g_5\\
&+ 319 g_1^2 g_2^2 g_3^2 g_4^3 g_5^2 + 461 g_1^2 g_2^2 g_3^2 g_4^2 g_5^2 g_6\big) + \mathcal{O}\big((\gamma^2)^7\big),\end{aligned} \tag{1}$$

II. SELF-CONSISTENT PARQUET CALCULATIONS

For realistic systems methods based on the notion of four-point vertex Γ might be a viable alternative. In this approach[1,2] the correlated self-energy part is given by:

$$\Sigma^c(18) = -\frac{\gamma^2}{2}\int V(1234) G(36) G(45) \Gamma(56,78) G(72)\, d(234567). \tag{2}$$

In full generality $\Gamma(12,34)$ can be obtained by solving a set of coupled Bethe-Salpeter equations in particle-particle ([12]) and particle-hole ([14] and [13]) channels known as parquet equations[3–5]. If only one such channel is considered one arrives at the T-matrix approximations known to complement the GW-approximation[6–10].

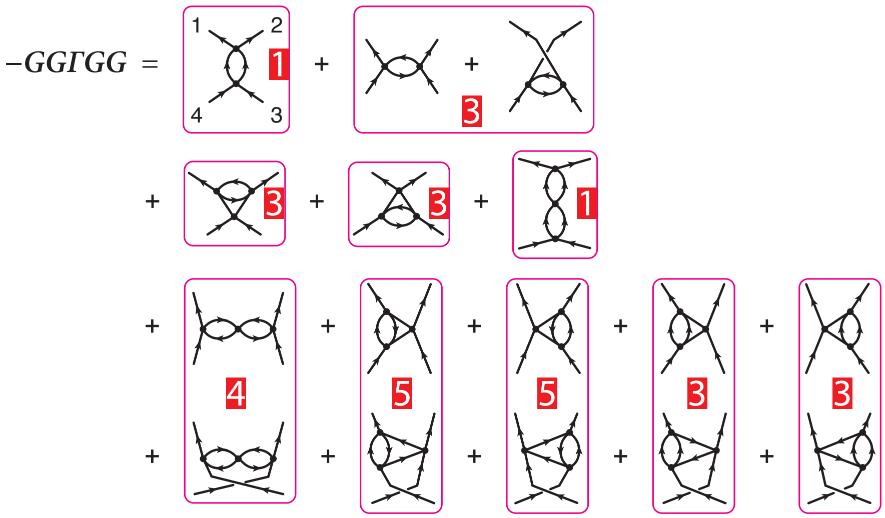

FIG. 1. Hugenholtz diagrams for the second and third order contribution to the two-particle Green's function. Each diagram results in a number (shown next to the diagram) of self-energy terms through the application of Eq. (2). Dots denote the anti-symmetrized Coulomb interaction. The first diagram and the second row of diagrams are non-simple in the particle-particle channel. They can be separated into two disconnected parts, such that one part contains the lines 1 and 2 and the other one the lines 3 and 4. Complementary of these diagrams are *simple* in the particle-particle channel ([12]). They can be further classified into the non-simple particle-hole diagrams in the [14] (third row) and [13] (fourth row) channels. Starting from the fourth order simple diagrams in all channels, i. e. [12], [13], and [14] appear.

---

a)yaroslav.pavlyukh@physik.uni-halle.de

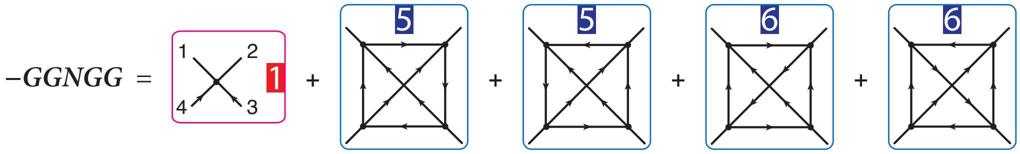

FIG. 2. Hugenholtz diagrams for the first and the fourth order diagrams which are simple in all channels. Each diagram results in a number (shown next to the diagram) of self-energy terms through the application of Eq. (2). Blue color indicates vertices omitted from our parquet solution.

Since the simplest T-matrix approximations lead to self-energies with fermionic loops (cf. Fig. 1(d) of Ref. 10), we consider here a more general class of diagrams (Fig. 1) resulting from the parquet calculations with only one simple four-vertex (Fig. 2). Explicitly, 22 and 714 diagrams of the 5th and 6th orders

$$\begin{aligned}\Delta\Sigma &= (\gamma^2)^5\big(4g_1^2g_3^3g_2^4 + 4g_1^2g_3^3g_4g_2^3 + 8g_1^2g_3^3g_4^2g_2^2 + 6g_1^2g_3^2g_4^2g_5g_2^2\big)\\ &+ (\gamma^2)^6\big(16g_1^2g_3^4g_2^5 + 8g_1^3g_3^3g_2^5 + 60g_1^2g_3^4g_4g_2^4 + 8g_1^3g_3^3g_4g_2^4 + 82g_1^2g_3^4g_4^2g_2^3 + 16g_1^3g_3^3g_4^2g_2^3 + 56g_1^2g_3^3g_4^2g_5g_2^3\\ &+ 12g_1^3g_3^2g_4^2g_5g_2^3 + 74g_1^2g_3^4g_4^3g_2^2 + 120g_1^2g_3^2g_4^3g_5^2g_2^2 + 148g_1^2g_3^3g_4^3g_5g_2^2 + 114g_1^2g_3^2g_4^2g_5^2g_6g_2^2\big) + \mathcal{O}\big((\gamma^2)^7\big). \qquad (3)\end{aligned}$$

need to be omitted because they are the offsprings of fourth- and fifth-order simple vertices. We performed *sc* calculations for the same values of parameters as on Fig. 5 of the main text with this self-energy and found no deterioration of the results as compared to full 6th-order expansion. Therefore, it can be expected that even a better description of higher order satellites is achieved if the parquet procedure is iterated further.

III. ELECTRON-BOSON MODEL IN AN EXCITED BOSONIC STATE

A. Electron propagators

A reference solution for the electron-boson model (Eq. (3) of the main text) can be obtained using the Feynman disentangling of operators[11]. It allows to express $g^{[n_b]}(\omega)$ in terms of the shifted $n_b = 0$ propagators $g^{[0]} \equiv g(\omega)$; the first few terms are:

$$g^{[1]} = (1-2a^2)g_0 + a^2(g_1 + g_{-1}), \qquad (4a)$$

$$g^{[2]} = (1-4a^2+3a^4)g_0 + 2(a^2-a^4)(g_1+g_{-1}) + \frac{1}{2}a^4(g_2+g_{-2}), \qquad (4b)$$

$$g^{[3]} = (1-6a^2+9a^4-\frac{10}{3}a^6)g_0 + (3a^2-6a^4+\frac{5}{2}a^6)(g_1+g_{-1}) + (\frac{3}{2}a^4-\frac{5}{6}a^6)(g_3+g_{-3}). \qquad (4c)$$

B. Electron self-energy

For a pure bosonic case $\langle b^\dagger b\rangle = 1$, the Wick theorem is not directly applicable. Therefore the electron self-energy cannot be computed using standard diagrammatic methods. Nonetheless, perturbative series can be obtained using the method described in Sec. III of the main text. In terms of bare electron propagators the self-energy $\Sigma^{[1]} = \bar\Sigma_r[g^{(0)}]$ reads:

$$\begin{aligned}\bar\Sigma_r[g] &= \gamma^2\left(g_{-1}+2g_1\right) + 6(\gamma^2)^2g_1^2g_2 + 6(\gamma^2)^3\left(3g_2^2g_1^3 + 4g_2^2g_3g_1^2\right) + 6(\gamma^2)^4\left(9g_2^3g_1^4 + 24g_2^3g_3g_1^3 + 16g_2^3g_3^2g_1^2 + 20g_2^2g_3^2g_4g_1^2\right)\\ &+ 6(\gamma^2)^5\big(27g_2^4g_1^5 + 108g_2^4g_3g_1^4 + 144g_2^4g_3^2g_1^3 + 120g_2^3g_3^2g_4g_1^3 + 64g_2^4g_3^3g_1^2 + 100g_2^2g_3^3g_4^2g_1^2 + 160g_2^3g_3^3g_4g_1^2\\ &+ 120g_2^2g_3^2g_4^2g_5g_1^2\big) + \mathcal{O}\big((\gamma^2)^6\big), \qquad (5)\end{aligned}$$

[1] J. P. Blaizot and G. Ripka, *Quantum theory and finite systems* (Cambridge, MA, 1986).

[2] R. van Leeuwen, N. E. Dahlen, and A. Stan, "Total energies from variational functionals of the Green function and the renormalized four-point vertex," Phys. Rev. B **74**, 195105 (2006).

[3] B. Roulet, J. Gavoret, and P. Nozières, "Singularities in the X-Ray Absorption and Emission of Metals. I. First-Order Parquet Calculation," Phys. Rev. **178**, 1072–1083 (1969).

[4] A. O. Gogolin, A. A. Nersesyan, and A. M. Tsvelik, *Bosonization and strongly correlated systems* (Cambridge University Press, Cambridge, U.K.; New York, NY, 1998).

[5] G. Rohringer, A. Valli, and A. Toschi, "Local electronic correlation at the two-particle level," Phys. Rev. B **86**, 125114 (2012).

[6]S. Nagano, K. S. Singwi, and S. Ohnishi, “Correlations in a two-dimensional quantum electron gas: The ladder approximation,” Phys. Rev. B **29**, 1209–1213 (1984).
[7]C. Verdozzi, R. W. Godby, and S. Holloway, “Evaluation of GW Approximations for the Self-Energy of a Hubbard Cluster,” Phys. Rev. Lett. **74**, 2327–2330 (1995).
[8]I. A. Nechaev and E. V. Chulkov, “Variational solution of the T-matrix integral equation,” Phys. Rev. B **71**, 115104 (2005).
[9]Z. Qian, “On-top pair-correlation function in the homogeneous electron liquid,” Phys. Rev. B **73**, 035106 (2006).
[10]M. P. von Friesen, C. Verdozzi, and C.-O. Almbladh, “Successes and Failures of Kadanoff-Baym Dynamics in Hubbard Nanoclusters,” Phys. Rev. Lett. **103**, 176404 (2009).
[11]G. Mahan, *Many-particle physics*, 3rd ed. (Kluwer Academic/Plenum Publishers, New York, 2000).
